# Unraveling CD69 signaling pathways, ligands and laterally associated molecules

**DOI:** 10.17179/excli2022-5751

**Published:** 2023-03-16

**Authors:** María Jiménez-Fernández, Hortensia de la Fuente, Pilar Martín, Danay Cibrián, Francisco Sánchez-Madrid

**Affiliations:** 1 Hospital Universitario de la Princesa, Universidad Autónoma de Madrid (UAM), Instituto de Investigación Sanitaria del Hospital Universitario de La Princesa (IIS-IP), 28006 Madrid, Spain; 2 Centro Nacional de Investigaciones Cardiovasculares (CNIC), 29029 Madrid, Spain; 3 CIBER de Enfermedades Cardiovasculares (CIBERCV), Instituto de Salud Carlos III, 28029 Madrid, Spain

**Keywords:** CD69, CD69-ligands, CD69-signaling, molecular signaling-pathway

## Abstract

CD69 is an early leukocyte activation marker involved in the regulation of the immune response. Initial *in vitro* studies evaluated its function using monoclonal antibodies until knock-out mice were developed. Subsequently, four ligands for CD69 have been identified, namely galectin-1, S100A8/S100A9 complex, myosin light chains 9 and 12, and oxidized low-density lipoproteins. In addition, several molecules are laterally associated with and regulated by CD69, including calreticulin and two transmembrane receptors, sphingosine-1-phosphate receptor (S1P1) and the heterodimeric amino acid transporter complex SLC7A5-SLC3A2 (LAT1-CD98). Recently, CD69 engagement has been shown to induce the expression of the immunoregulatory receptor programmed cell death-1 (PD-1) in T cells. The molecular signaling induced by CD69 has been explored in different scenarios and cell types. This review provides a perspective on the molecular pathways, ligands and cellular functions known to be regulated by CD69.

## Introduction

CD69 is a type-II transmembrane receptor of the C-type calcium-dependent lectin superfamily (López-Cabrera et al., 1993[40]; Ziegler et al., 1993[87]). Four different groups described this molecule in the late 80s as an early molecule rapidly induced on human leukocytes upon activation (Hara et al., 1986[28]; Cosulich et al., 1987[12]; Cebrián et al., 1988[6]; Lanier et al., 1988[37]). CD69 is a glycosylated homodimeric receptor formed by two disulphide-linked chains of 28 and 32 KDa (Testi et al., 1989[77]; López-Cabrera et al., 1993[40]). These size differences reside in the number of N-glycan chains, as human CD69 has two N-glycosylation sites, the consensus motif (amino acids (AA) 166-168) and the atypical one (Vance et al., 1997[84]). Both chains are constitutively phosphorylated on serine residues in the intracellular portion (Testi et al., 1988[76]), which also contains the N-terminal region with a short cytoplasmatic tail (1-40 AA). As a member of the C-type lectin superfamily, the extracellular C-terminal portion contains the CTLD domain. CD69 is related to the natural killer (NK) cell receptor family, as the gene locus is located in the NK gene cluster on chromosomes 6 and 12 in mice and humans, respectively (López-Cabrera et al., 1993[40]). 

This receptor is rapidly induced in B and T lymphocytes, NK cells, monocytes, macrophages, neutrophils, and eosinophils after different activation stimuli (Table 1(Tab. 1); References in Table 1: Borrego et al., 1999[3]; Cebrián et al., 1988[6], 1989[5]; Conde et al., 1996[10]; D'Ambrosio et al., 1993[14]; De Maria et al., 1994[17]; Foerster et al., 2002[21]; Gavioli et al., 1992[23]; Moretta et al., 1991[50]; Nakamura et al., 1989[51]; Pisegna et al., 2002[55]; Ramírez et al., 1996[59]; Sánchez-Mateos et al., 1989[63]; Sancho et al., 2000[65]; Santis et al., 1992[66]; Testi et al., 1989[78], 1990[79], 1992[80]; Tugores et al., 1992[82]; Walsh et al., 1996[85]; Zingoni et al., 2000[88]). Since a wide variety of stimuli such as small activation molecules, anti-CD3 engagement, cytokines, lipopolysaccharide, and heat shock, among others, can induce its early expression at the surface membrane, it has been used as a classic lymphocyte activation marker. CD69 is detected within hours of stimulation because it is pre-formed in the cytoplasm of resting cells and the mRNA and protein synthesis is immediately triggered (Risso et al., 1991[60]). It is constitutively expressed by platelets and a small proportion of cortical thymocytes, where it regulates positive thymocyte selection (Testi et al., 1988[76], 1990[79]; Hare et al., 1999[29]). In addition, positively selected CD69^hi^TCR-αβ^hi^ double positive thymocytes developed into natural Foxp3^+ ^regulatory T (Treg) cells (Martín-Gayo et al., 2010[45]). Foxp3^+ ^CD69^+^ Treg cell subset expresses high levels of immune suppression molecules such as CTLA-4, ICOS, GITR and CD38, enhanced TGF-β production and tolerogenic potential (Cortés et al., 2014[11]). In addition, CD69 is expressed by most tissue-resident memory T cell (TRM) subsets, and thus, along with CD103, is commonly used as their identification marker (Steinbach et al., 2018[73]). Remarkably, CD69 is expressed by *in vivo* activated lymphocytes, infiltrating inflammatory foci in chronic autoimmune inflammatory diseases such as rheumatoid arthritis, chronic viral hepatitis and many others (González-Amaro et al., 2013[25]).

In this review, we provide insight into CD69-mediated molecular signaling that has been described over the years and its possible implications.

## CD69 Antibody-Mediated Signaling Pathway

The use of specific monoclonal antibodies against CD69 provided the first tool to characterize the molecular signaling induced by this receptor. The effects of CD69 engagement in different cell types are summarized in Table 1(Tab. 1), with T-lymphocytes being the most studied cell. Several anti-CD69 antibodies induce CD69 internalization thus blocking the interaction with putative ligands. In combination with PKC-stimulating agents such as phorbol esters, it was concluded that anti-CD69 antibodies trigger lymphocyte proliferation by inducing the expression of IL-2 and CD25 and increasing TNF-α secretion (Cebrián et al., 1988[6], 1989[5]; Nakamura et al., 1989[51]; Santis et al., 1992[66]). Moreover, cross-linking of CD69 by secondary antibodies induces an increase in intracellular calcium concentration in T cells, which along with PKC activation also induces IFN-γ expression (Testi et al., 1989[78]). Anti-CD69 antibody-induced transcriptional response relies on AP-1 activity with c-fos involvement after CD69 stimulation and calcium mobilization-mediated activation of Nuclear Factor of Activated T Cells 1 (NFAT) (Tugores et al., 1992[82]; D'Ambrosio et al., 1993[14]). Aside, functional effects have been described in other cell types such as B cell proliferative response (Sánchez-Mateos et al., 1989[63]). In platelets, CD69 engagement induces Ca^2+^ influx, activating phospholipase A2 (PLA2) and arachidonic acid metabolism, leading to aggregation and degranulation (Testi et al., 1990[79], 1992[80]). A similar molecular response was observed in human monocytes, in addition to strong nitric oxide (NO) production (De Maria et al., 1994[17]). Ca^2+^ mobilization, and expression of intercellular adhesion molecule-1 (ICAM-1), CD25 and TNF-α were also triggered in pre-activated NK cells by anti-CD69 antibodies (Borrego et al., 1999[3]). The binding of CD69 by anti-CD69 antibodies triggers cytolytic activity in NK cells and γδ T cells but not αβ T cells, in an extracellular signal-regulated kinase (ERK) enzymes-dependent manner (Moretta et al., 1991[50]; Zingoni et al., 2000[88]). In addition, antibodies induce granule exocytosis in neutrophils and mast cells activation (Gavioli et al., 1992[23]; Sancho et al., 2000[65]). Interestingly, anti-CD69 antibodies blocked IL-1β production of THP-1 cells cultured with Jurkat cells and also inhibited the ability of T cells to activate macrophages (Manié et al., 1993[42]; Mcinnes et al., 1997[48]). Taken together, these effects of anti-CD69 antibodies indicated a role in cell activation, which would not be unexpected given their rapid membrane expression in response to multiple stimuli. However, mice lacking CD69 showed no differences in antigen-dependent T cell activation, except in B cell development (Lauzurica et al., 2000[38]). Bone marrow analysis showed higher B220^hi^IgM^neg^ pre-B cells and a modest increase in the immunoglobulin (Ig) G2a and IgM production, as T-dependent and T-independent antigens response was found in the absence of CD69 (Lauzurica et al., 2000[38]). The implications of the regulation exerted by CD69 over B cell functions have not been explored in more detail. It was also shown that anti-CD69 antibodies induce apoptosis in eosinophils and macrophages and THP1 cells in presence of LPS stimulation (Ramírez et al., 1996[59]; Walsh et al., 1996[85]; Foerster et al., 2002[21]). 

Taken together, these antibody-induced effects indicated that CD69 exerts a major positive role in immune cell activation. However, when the role of CD69 was evaluated using animal models of chronic inflammation *in vivo*, its important role as a negative immune regulatory molecule was revealed (González-Amaro et al., 2013[25]). CD69-deficient mice showed a significant susceptibility to collagen-induced arthritis (CIA), allergic asthma, contact dermatitis, autoimmune myocarditis and colitis induced by CD45Rb^+^ CD4^+^ T cell transfer (Sancho et al., 2003[64]; Cruz-Adalia et al., 2010[13]; Martín et al., 2010[44]; Radulovic et al., 2012[57]). Administration of blocking CD69-antibodies also increased cytokine release by T cells and exacerbated inflammatory responses in animal models (Cruz-Adalia et al., 2010[13]; Martín et al., 2010[44]). Nevertheless, CD69 can also play an inflammatory role in tissue inflammation, highlighting the effect of CD69 on T cell migration and retention in the tissue (Hasegawa et al., 2013[30]; Cibrián et al., 2016[8]). Hence, the complexity of CD69-mediated regulation of the immune response involves not only its possible intracellular signaling pathway but also interaction with membrane partners and ligands in tissues. The detailed signals transmitted through the extracellular or transmembrane region vs cytoplasmic tail of CD69 have not yet been fully elucidated.

## CD69 Ligands

**Figure 1:**
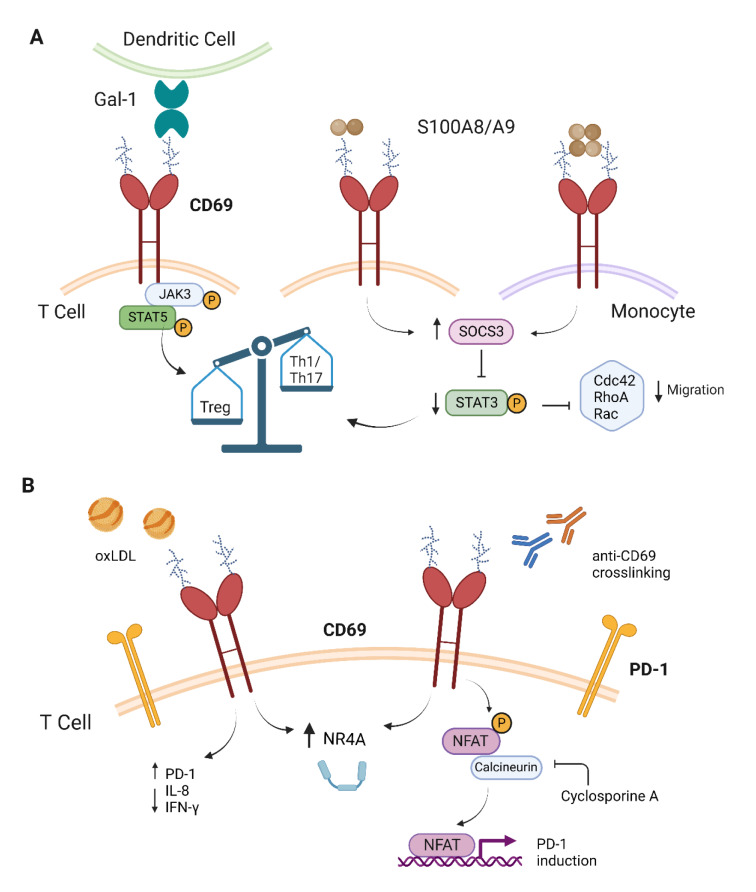
Molecular signaling induced by CD69 engagement. A) Glycosylation-dependent CD69 ligand-mediated signalling. Binding of Gal-1 of human monocyte-derived dendritic cells to CD69 increases the Treg cell differentiation mostly mediated by JAK3/STAT5 activation. S100A8/S100A9 dimers bind to CD69 on T cells, enhancing SOCS3 expression, which reduces STAT3 phosphorylation and promotes Treg cell development. S100A8/S100A9 tetramers bind to CD69 on monocytes, activating STAT3 modulation through SOCS3 expression and reducing the activity of small GTPases, such as Cdc42, RhoA and Rac, and thus monocyte migration. B) OxLDL-CD69 binding and crosslinking of anti-CD69 antibody induce PD-1 expression. The binding of oxLDL to CD69 induces the expression of the nuclear receptors NR4A, the levels of PD-1 and decreases IL-8 and IFN-γ expression. Crosslinking of anti-CD69 antibodies induces the NFAT activation that leads to PD-1 expression. Cyclosporine A inhibits the PD-1 induction by CD69 engagement. This figure was created with BioRender.com.

For years, attempts have been made to identify potential ligands of CD69 to better understand its function. So far, four ligands have been described and studied to varying degrees of depth. Galectin-1 (Gal-1) was the first protein described, followed by S100A8/S100A9 complex, myosin light chain (Myl) 9 and Myl12 and oxidized low-density lipoprotein (oxLDL) (Figure 1A(Fig. 1)) (de la Fuente et al., 2014[16]; Lin et al., 2015[39]; Hayashizaki et al., 2016[31]; Tsilingiri et al., 2019[81]). The multiple CD69 interacting proteins described suggest that there is a broad context in which this receptor may play its functional roles.

The binding of the first ligand described, the protein Gal-1, was characterized by mass spectrometry, surface plasmon resonance (SPR) and binding assays (de la Fuente et al., 2014[16]). Gal-1 is a soluble protein secreted by many cell types that can bind to glycosylated membrane proteins and regulate inflammatory responses (Sundblad et al., 2017[74]). *In vitro* studies showed the interaction of Gal-1 on human monocyte-derived dendritic cells with CD69 expressed by CD4^+^ T cells in a carbohydrate-dependent manner (de la Fuente et al., 2014[16]). The Gal-1-CD69 binding limits Th17 differentiation of human and mouse CD4^+^ T cells (de la Fuente et al., 2014[16]). Thus, the binding of Gal-1 to CD69 is thought to contribute to the regulation of Th17 differentiation. 

The second ligand identified by immunoprecipitation and mass spectrometry was the calcium-binding S100A8/S100A9 complex, also known as calprotectin (Figure 1A(Fig. 1)). The authors showed that the binding of S100A8/S100A9 derived from human peripheral mononuclear cells to CD69 was carbohydrate-dependent and favored Treg differentiation with reduced levels of phospho-STAT3 and high SOCS3 expression (Lin et al., 2015[39]). PMA-activated hPBMCs treated with S100A8/S100A9 displayed enhanced TGF-β and reduced IL-4 secretion compared to small hairpin RNA targeting CD69 (shCD69) T cells, which also showed an increment in IL-17A (Lin et al., 2015[39]). As with Gal-1, this ligand is not specific for CD69 because the S100A8/S100A9 complex recognizes carboxylated glycans (Srikrishna et al., 2001[72]). However, both ligands can shape the Th17/Treg phenotype through *in vitro* binding to CD69. The role of this ligand binding to CD69 on monocytes has recently been demonstrated. Specifically, by direct binding experiments employing the quartz crystal microbalance with dissipation (QCM-D) technique, the authors probed that CD69 binds to S100A8/S100A9 tetramers on monocytes with a dissociation constant in the low nanomolar range (Russo et al., 2022[61]). This interaction controls the monocyte migratory capacity through the activation of SOCS3, which reduces STAT3 phosphorylation and regulates the small GTPases activity (Russo et al., 2022[61]). Hence, CD69 deficient monocytes showed higher levels of phosphorylated STAT3 as well as reduced levels of SOCS3 (Russo et al., 2022[61]), pointing to dysregulation of this molecular pathway intrinsic to the absence of CD69. Thus, in inflammation sites where monocytes were activated, the binding of S100A8/S100A9 tetramers to CD69 would be regulating their inflammatory state as a counteracting effect. Moreover, this complex would be able to induce Treg differentiation to favor the resolution of inflammation. 

Myosin light chain (Myl) 9 and Myl12 were also reported to be functional ligands of CD69 accounting for the migration of activated T helper cells (Hayashizaki et al., 2016[31]). The ligand was identified by affinity purification employing the GST-His-CD69 extracellular domain as bait and subsequently mass spectrometry analysis. Using different mutants of Myl9, the authors probed that CD69 binding occurs through the N-terminal region of Myl9 and depends on the positive charges of lysine residues (Hayashizaki et al., 2016[31]). In this context, blocking with anti-CD69 or anti-Myl9/12 antibodies ameliorated the inflammation in allergic airway inflammatory models (Hayashizaki et al., 2016[31]). The authors propose that Myl9/12 provides a platform to attract and retain CD69^+^ T cells; however, whether this ligand regulates the T cell differentiation process or induces CD69-mediated intracellular signaling remains to be elucidated. 

The most recent ligand described for CD69 in CD4^+^ T cells is oxLDL (Figure 1B(Fig. 1)). The C-type lectin-like domains of CD69 and oxLDL receptor 1 (LOX-1) share similar structural features necessary for oxLDL binding and both genes are closely located on the same chromosome (Tsilingiri et al., 2019[81]). OxLDL-CD69 binding induces the internalization of the receptor and reduces the levels of IL-8 and IFN-γ in T lymphocytes (Tsilingiri et al., 2019[81]). Moreover, *in vitro* differentiation assays with human CD4^+^ T cells exposed to oxLDL showed a reduction of Th17 and Th1 response and an increased expansion of Treg (Tsilingiri et al., 2019[81]). Blocking this interaction with anti-CD69 antibodies proved the specificity of CD69 in mediating these phenotypes. The main source of IFN-γ during the atheroma plaque formation are Th1 cells, which are regulated by NF-κB activation (Hansson and Libby 2006[27]; Oh and Ghosh 2013[54]). Whether CD69 may control NF-kB activity has not been properly explored. In addition, oxLDL-CD69 binding enhanced the expression of NR4A transcription factors, specifically NR4A3 and NR4A1 (Tsilingiri et al., 2019[81]). NR4A3 and NR4A1 belong to the NR4A orphan nuclear receptors, controlling Treg cell development and maintenance (Sekiya et al., 2013[68]; Odagiu et al., 2021[53]). Interestingly, the antigen activation of B cell receptor (BCR) on B lymphocytes induces primary response genes (PRGs) such as CD69, but also NR4A1 and NR4A3 (Tan et al., 2020[75]). In the absence of NR4A1, CD69 and some other PRGs enhance their expression indicating a negative feedback between these two molecules that does not happen in NR4A3 knock-out B cells (Tan et al., 2020[75]). Thus, the induction of NR4A nuclear receptors through the CD69 engagement could be regulating the CD69 expression itself. Lack of CD69 in the lymphoid compartment of *Ldlr*^-/- ^ mice fed a high-fat diet promotes accelerated development of atheroma plaques, consistent with enhanced Th17 responses in the para-aortic lymph nodes and increased Th17/Treg cell ratio in the blood (Tsilingiri et al., 2019[81]).

Recently, oxLDL binding to CD69 has been shown to induce the programmed cell death 1 (PD-1) expression on human CD4^+^ T cells, mostly through NFAT-mediated activation (Jiménez-Fernández et al., 2022[32]). Molecular cross-linking of CD69 with monoclonal antibodies enhanced NR4A nuclear receptors and PD-1 expression and the inhibition of NFAT with cyclosporine A impaired the enhancement of NR4A3 and PD-1 (Jiménez-Fernández et al., 2022[32]). Higher CD69, NR4A3 and PD-1 levels were found in inflamed abdominal aortic samples from patients with higher hyperlipidemia, which reinforces the relevant role of these molecules in the pathology progression. The role of PD-1 in mediating T cell-induced atherosclerotic injury has been previously characterized. Hypercholesterolemic *Ldlr* deficient and *Pd1 *double knockout mice developed larger atherosclerotic lesions with increased CD4^+^, CD8^+^ and macrophage infiltration and higher serum TNF-α levels (Bu et al., 2011[4]). The results were also confirmed with blocking anti-PD-1 antibody and *Ldlr*^−/−^ and *Pd-l1/l2*^−/−^ bone marrow chimeras. Recently, agonistic PD-1 antibody treatment diminished the atherosclerosis development in *Ldlr*^−/−^ mice, enhancing atheroprotective B and CD4^+^ T cell responses (Grievink et al., 2021[26]). The PD-1 regulation through CD69-oxLDL could contribute to the exacerbated phenotype observed in a previous atherosclerosis model in the bone marrow (BM) chimeric *Ldlr*^−/−^ mice deficient for CD69 only in the lymphoid compartment and also in asymptomatic individuals with subclinical atherosclerosis (Tsilingiri et al., 2019[81]).

## CD69 Cytoplasmic Tail Interaction and CD4+ T Cell Differentiation Programs

Numerous studies have shown that the absence of CD69 leads to an exacerbated form of inflammatory diseases *in vivo*, using mouse models (González-Amaro et al., 2013[25]). The study of the role of CD69 in the differentiation of T helper lineages revealed enhanced Th17 responses in mice lacking CD69, whereas Th1 and Th2 responses were unaltered (Martín et al., 2010[43]). Pull-down assays with the cytoplasmic tail of CD69 showed its association with JAK3/STAT5 suggesting regulation of this signaling pathway (Figure 1A(Fig. 1)). Accordingly, CD69 deficient cells displayed impaired STAT5 phosphorylation under Th17 differentiation conditions indicating a defect in the JAK3 signaling. JAK3/STAT5 signaling inhibition in CD69 deficient T cells favors dimerization and translocation of STAT3 to the nucleus, inducing RORγt expression and consequently a Th17 differentiation bias (Martín et al., 2010[43]). The interaction of Gal-1 with CD69 potentially regulates this mechanism to negatively control Th17 differentiation. in mouse and human CD4 T cells (de la Fuente et al., 2014[16]). The regulation of the JAK3/STAT5 pathway by CD69 has also been studied in the development of Thymus-derived regulatory T (tTreg) cells (Sánchez-Díaz et al., 2017[62]). CD69-deficient tTreg cells have impaired STAT5 phosphorylation as well as reduced levels of *bic*/miR-155 and higher expression of its downstream target molecule SOCS-1, a repressor for Treg differentiation (Sánchez-Díaz et al., 2017[62]). Blocking CD69 with monoclonal antibodies, that downregulate its membrane expression, also impairs STAT5 phosphorylation, reduces the levels of miR-155, thereby promoting SOCS-1 inhibition (Sánchez-Díaz et al., 2017[62]). This study highlights the role of CD69 as a key regulator of mir-155 and SOCS-1 expression during the generation and homeostasis of tTreg cells.

## CIS-Molecular Association of CD69 and their Functional Implications

By using immunoprecipitation and direct protein sequencing techniques, Vance et al., identified the association of CD69 with an N-terminal fragment of calreticulin (CALR) derived from the cell surface of PMA-activated human peripheral blood mononuclear cells (PBMCs) (Vance et al., 2005[83]). CALR is a Ca^2+^-binding protein from the endoplasmic reticulum responsible for the proper folding of proteins which can be also exposed on the surface membrane and have multiple additional functions (Fucikova et al., 2020[22]). B lymphocytes were identified as the main cell subset expressing both CD69 and CALR and thus the authors proposed that this association occurs laterally at the cell surface (Vance et al., 2005[83]). However, the nature of this interaction and its possible functional role in T cell activation and function have not been fully explored. 

**Figure 2:**
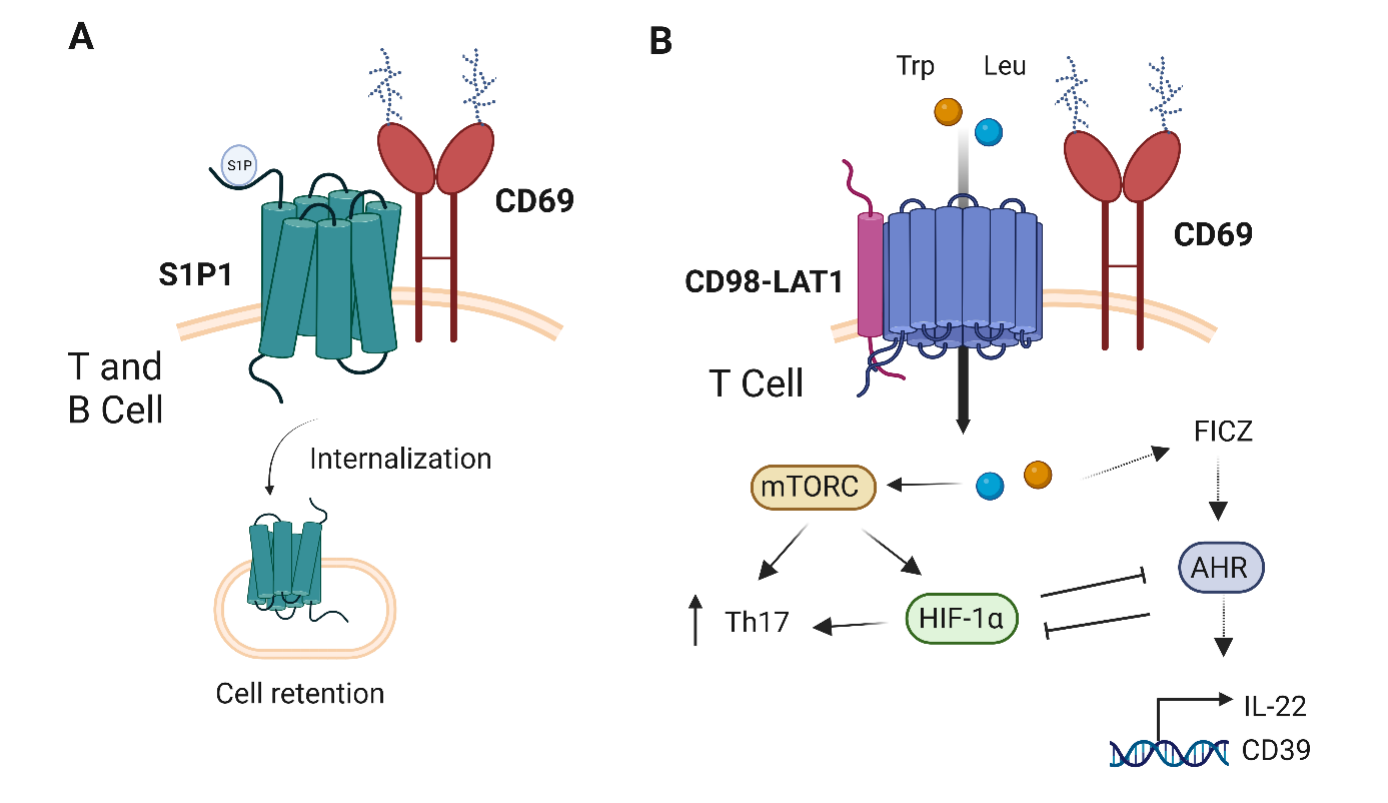
Lateral association of CD69 with transmembrane receptors. A) CD69 is associated with S1P1 on T and B cells which leads to downmodulation and degradation of S1P1, and impairs cell migration to S1P-dependent chemotaxis. B) CD69 interacts with the amino acid transporter SLC7A5-SLC3A2, also known as LAT1-CD98, stabilizing the expression of SLC7A5 on the T cell membrane. This interaction favors the amino acid uptake, increasing leucine (Leu) transport that activates mTORC and promotes Th17 cell differentiation. HIF-1α is also stimulated by mTORC and mediates Th17 differentiation. CD69 increases the uptake of tryptophan (Trp) and the Trp-derived metabolite FICZ, which is an AHR agonist. AHR activation induces the transcription of IL-22 and CD39 mRNA. AHR modulates HIF-1α levels and vice versa, which can also impact in the Th17 cell differentiation program. This figure was created with BioRender.com.

A well-established function of CD69 is the control of lymphocyte migration through the negative regulation of sphingosine 1 phosphate receptor (S1P1) expression on the membrane (Figure 2(Fig. 2)). The S1P1 is involved in regulating the lymphocyte egress from the thymus and the lymphoid organs to the circulation in response to the sphingosine-1-phosphate (S1P) chemoattractant gradient (Matloubian et al., 2004[47]). After IFNα/β signaling, CD69 is expressed by T cells and forms a complex with S1P1 causing its internalization and thus blocking the lymphocyte egress (Shiow et al., 2006[69]). The nature of this interaction was subsequently described as involving helix 4 of S1P1 and transmembrane and membrane proximal regions of CD69, whereas the CTLD domain enhances the interaction between both receptors (Bankovich et al., 2010[1]). Likewise, the S1P1/CD69 axis has also been shown to control the migration of mouse skin dendritic cells (DC) to lymph nodes (LNs) in a contact sensitization model. In the absence of CD69, the ability of skin DCs to migrate to the LNs is increased as evidenced by subcutaneous transfer of deficient cells and two-photon microscopy analysis (Lamana et al., 2011[36]). Chemotaxis to S1P and expression of S1P1 is higher in *Cd69^-/-^* DCs and the use of S1P analogues (SEW2871 and FTY720) impaired the DC migration from the skin to peripheral LNs (Lamana et al., 2011[36]). Thus, CD69 expression controls the retention of the cells in the lymphoid organs, allowing them to differentiate and activate properly before exiting into circulation. CD69 also controls monocyte migratory capacity through the regulation of pSTAT3 levels by SOCS3 (Lin et al., 2015[39]; Russo et al., 2022[61]), thereby CD69 has two pathways controlling cell migration. Better characterizing the intracellular signaling of CD69 ligands could provide insights into CD69-dependent regulation of SOCS3 and Th cell differentiation. Nevertheless, CD69 knockout mice have normal thymocyte development pointing out that this function is not exclusive to CD69 and it might be controlled by other molecules (Lauzurica et al., 2000[38]). Most recently, one exception to this was reported, in which the expression of CD69 on CD24^+^PLZF^hi^ innate precursors was essential to retain them in the thymus and to develop into mature NKT2 cells (Kimura et al., 2018[33]). However, this regulation was partially independent of S1P1, since its deletion did not completely restore the NKT2 cell differentiation, suggesting that CD69 plays a role beyond cell retention through the control of S1P1 (Kimura et al., 2018[33]). Further experiments involving the CD69 ligands-mediated signaling, or a better understanding of its intrinsic signaling, may shed light on how CD69 regulates NKT2 cell generation. 

In addition to controlling the cell egress from the thymus and the lymph nodes, the role of the CD69-S1P1 axis was also studied in the regulation of lymphocyte egress from the BM. Blockade of CD69 with monoclonal antibodies induced a rapid mobilization of large numbers of BM leukocytes, which is inhibited by using the agonist of S1P1 FTY720 (Chiba 2005[7]; Notario et al., 2018[52]). The anti-CD69 treatment also promoted the proliferation of hematopoietic stem and progenitor cells and the increase of mTOR, p70S6K, S6 and 4E-BP1 phosphorylation (Notario et al., 2018[52]). Interestingly, cell mobilization was inhibited by rapamycin, indicating that CD69 acts through mTOR as previously described and discussed below (Cibrián et al., 2016[8]). However, in this context, it has not been thoroughly studied whether this upregulation of mTOR when using an anti-CD69 antibody is due to an agonistic effect or the loss of CD69 expression on the membrane. 

The role of the S1P1 regulation through CD69 has also been studied on TRM, where CD69 is widely expressed. In this context, skin CD8^+^ effector cells upregulate membrane CD69 in response to multiple signals, which represses S1P1 expression, allowing its retention and local memory formation (Skon et al., 2013[71]; Mackay et al., 2015[41]). It is noteworthy that CD69 has also been implicated in the control of CD4^+^ T cell migration independently of S1P1. CD69 deficient cells have enhanced expression of the chemokines CCL-1, CXCL-10 and CCL-19 on T cells from different inflammatory bowel disease murine models (Radulovic et al., 2013[58]). The administration of neutralizing antibodies against these chemokines improved the outcome of CD69 deficient mice under dextran sodium sulphate (DSS)-induced colitis (Radulovic et al., 2013[58]).

Additionally, CD69 also associates on cis with SLC7A5-SLC3A2 on the T cells plasma membrane (Figure 2(Fig. 2)) (Cibrián et al., 2016[8]). This association appears to stabilise or control the expression of SLC7A5 on the surface membrane of activated CD4^+^ T cells, regulating the amino acid uptake (Cibrián et al., 2016[8]; Cibrián and Sánchez-Madrid, 2017[9]). SLC7A5-SLC3A2 transporter mediates the high-affinity antiport of tryptophan, phenylalanine, leucine and histidine (Scalise et al., 2018[67]). Specifically, CD69-deficient CD4^+ ^and γδ T cells had less uptake of L-Trp, L-Phe and L-Leu and the antibody-induced internalization of CD69 prevented L-Trp and L-Phe capture in wild-type CD4^+^ cells (Cibrián et al., 2016[8]). The amino acids uptake mediated by SLC7A5 modulates the activation of the mammalian target of rapamycin complex 1 (mTORC1) (Sinclair et al., 2013[70]). This serine/threonine kinase regulates multiple T cell functions but also promotes Th17 differentiation and suppresses the generation of FoxP3 regulatory T cells (Kopf et al., 2007[34]). *In vitro* CD69-deficient differentiated Th17 cells showed impaired phosphorylation of mTORC1 downstream kinases S6 and 4E-BP1 after TCR engagement, indicating that CD69 is required for the proper performance of the mTORC1 signaling pathway (Cibrián et al., 2016[8]). Aryl hydrocarbon receptor (AHR)-dependent responses were also evaluated in CD69-deficient mice in a model of psoriasis induced by IL-23, as these mice developed mild psoriasis and showed reduced IL-22 and STAT3 levels. AHR activates the RORγt-mediated IL-22 transcription in Th17 cells and the binding of this cytokine to its receptor induces the activation and phosphorylation of STAT3 in CD4 T cells and group 3 innate lymphocytes (ILC3) (Glal et al., 2018[24]; Zenewicz, 2018[86]). The metabolite derived from L-Trp 6-formylindolo [3,2-b] carbazole (FICZ) is an AHR agonist (Fernández-Gallego et al., 2021[20]). FICZ uptake was higher in CD69^+^ T cells and LAT1-dependent, supporting the regulation of AHR signaling by CD69 and IL-22 secretion in dermal γδ T cells (Cibrián et al., 2016[8]). In addition to binding the SLC7A5-SLC3A2 complex, differential proteomic analysis revealed a possible association of CD69 with other amino acid transporters such as GTR1 and MOT1 (Cibrián et al., 2016[8]). Further studies will determine whether CD69 could regulate the membrane expression of other amino acid transporters and their impact on cellular metabolism.

In addition to controlling IL-22 secretion, AHR is a ligand-activated transcriptional factor capable of regulating Treg cell development and Th17 cell differentiation in a ligand-dependent manner (Quintana et al., 2008[56]). Specific binding of FICZ to AHR promotes Th17 differentiation to the detriment of Treg cell development, increasing disease severity in a model of experimental autoimmune encephalomyelitis (Quintana et al., 2008[56]). AHR forms a complex with the AHR nuclear translocator (ARNT or HIF-1β) and controls HIF-1α levels, as HIF-1α regulates AHR degradation, thereby affecting the differentiation of type 1 regulatory T cells (Tr1) (Mascanfroni et al., 2015[46]). Extracellular ATP and hypoxia induce the inactivation of AHR via HIF-1α, inhibiting Tr1 differentiation and, conversely, CD39 promotes AHR activation by degrading ATP (Mascanfroni et al., 2015[46]). Interestingly, HIF-1α mediates Th17 differentiation through RORγt and IL-17 activation, after mTOR stimulation (Dang et al., 2011[15]). Moreover, CD69 is directly regulated by HIF-1α, which can bind to a hypoxia response element (HRE) at the locus of human CD69, as demonstrated by ChIP experiments (Labiano et al., 2017[35]). Thus, CD69 is involved in the regulation of the immune response through the regulation of AHR, possibly with HIF-1α implication and affecting the metabolism through the regulation of amino acid uptake by SLC7A5 (Cibrián and Sánchez-Madrid, 2017[9]). A recent report has also described that CD69^+^ Tregs, through an AHR-dependent mechanism, express high levels of membrane CD39 in hypoxic conditions after myocardial infarction (MI). CD39-mediated conversion of extracellular ATP to adenosine by CD69^+^ Tregs cells induced apoptosis of proinflammatory γδT cells infiltrating ischemic myocardium (Blanco-Domínguez et al., 2022[2]). Mice lacking CD69 and patients with lower CD69 expression of Tregs showed a worse prognosis after acute MI (Blanco-Domínguez et al., 2022[2]). 

## CD69 as an Immune Checkpoint: Relevance of the CD69-Mediated PD-1 Regulation

Recently, the engagement of CD69 by its ligand oxLDL has been shown to induce the expression of the immunoregulatory molecule PD-1 on human CD4^+^ T cells (Jiménez-Fernández et al., 2022[32]). *Cd69* deficient mice showed enhanced T cells and NK-mediated anti-tumor responses due to increased IFNγ secretion and cytolytic activity, respectively (Esplugues et al., 2003[18]). Downregulation of CD69 by antibody treatment also improved antitumor activity in WT mice (Esplugues et al., 2003[18]). This observation was validated by an independent group using a different *Cd69*-deficient germline and a different antibody treatment. Interestingly, these authors identified a higher frequency of exhausted CD8^+^ tumor-infiltrating lymphocytes (TILs), both PD1^hi^Tim^3-^ and PD1^hi^Tim^3+^, in the WT mice than in CD69-deficient mice (Mita et al., 2018[49]). The percentage of CD69^+^ cells was elevated in both populations and more evident in the PD1^hi^Tim^3+^ cells, classified as extremely exhausted cells (Mita et al., 2018[49]). Moreover, the anti-CD69 therapy resulted in higher CD4^+^ and CD8^+^ TILs with lower PD1^hi^Tim^3+ ^exhausted CD8^+^ T cells, but the mechanism was not identified (Mita et al., 2018[49]). Overall, these studies suggest a key role of CD69 in T-cell exhaustion through the regulation of PD-1 expression, conceivably by the activation of CD69-mediated signaling by known or unknown ligands in the tumor microenvironment.

The relationship between CD69 and PD-1 expression and the exhaustion program has also been shown in human atherosclerotic disease. Specific immune dysregulation was observed by an in-depth study combining mass-cytometry (CyTOF), cellular indexing of transcriptomes and epitopes by sequencing (CITE-seq) and single-cell RNA-seq, of blood and carotid artery plaques of symptomatic or asymptomatic patients (Fernandez et al., 2019[19]). T cells were enriched in the plaques compared to the blood, with the majority being effector memory (EM) T cells with high CD69 and PD-1 expression (Fernandez et al., 2019[19]). The authors concluded that T cells show various states of activation along with exhaustion in the atheroma plaque. Thus, CD69 may play a dual role in atherosclerosis development and possibly other pathologies through modulation of PD-1 levels and the control of T cells exhaustion program (Fernandez et al., 2019[19]; Tsilingiri et al., 2019[81]; Jiménez-Fernández et al., 2022[32]). 

## Concluding Remarks

In this review, we have described that the CD69 receptor acts as a regulator of activation, migration, retention, metabolism, and T cell exhaustion, and also as a regulator of pro-inflammatory Th17 responses. Different ligands bind to CD69 modulating several signaling pathways, therefore, there is a wide range of scenarios by which this molecule can regulate the function of immune cells. Importantly, the expression of CD69 ligands in different inflammatory settings can shape the CD69-mediated function on immune cells. Lateral association with membrane receptors, such as S1P1 and SLC7A5-SLC3A2 amino acid transporter, can also control the complex effects that CD69 exerts on immune cells. It is therefore conceivable that novel ligands will be identified in the future, as well as novel lateral associated proteins, potentially leading to new mechanisms of immune cell regulation mediated by CD69. It will also be interesting to further explore whether CD69 associates with additional intracellular signalling molecules, when it is expressed on the membrane, even in the absence of ligands, or after its internalization, to better understand the complex role of CD69 as an immunoregulatory molecule.

## Notes

Danay Cibrián and Francisco Sánchez-Madrid (Hospital Universitario de la Princesa, Universidad Autónoma de Madrid (UAM), Instituto de Investigación Sanitaria del Hospital Universitario de La Princesa (IIS-IP), IIS-IP, c/ Diego de León, 62, 28006 Madrid, Spain; E-mail: fsmadrid@salud.madrid.org) contributed equally as corresponding author.

## Declaration

### Conflict of interest

The authors have no conflict of interest to declare.

### Consent for publication 

Consent to publish has been received from all participants.

### Acknowledgments

This work was supported by grant P2022/BMD7209-INTEGRAMUNE from the Comunidad de Madrid to FS-M and PM, a grant from “La Caixa” Banking Foundation (HR17-00016) to FS-M; the Spanish Ministerio de Ciencia e Innovación (PDC2021-121719-I00 and PID-2020-120412RB-I00 to FS-M); and grants from the Instituto de Salud Carlos III (PI21/01583 to HF; PI22/01842 to DC; and PI22/01759 to PM). M. Jiménez-Fernández is supported by Formación de Personal Investigador Severo Ochoa (FPI-SO) Program (PRE2019-087941). The CNIC is supported by the Instituto de Salud Carlos III (ISCIII), the Ministerio de Ciencia e Innovación and the Pro CNIC Foundation and is a Severo Ochoa Center of Excellence (SEV-2015-0505).

## Figures and Tables

**Table 1:**
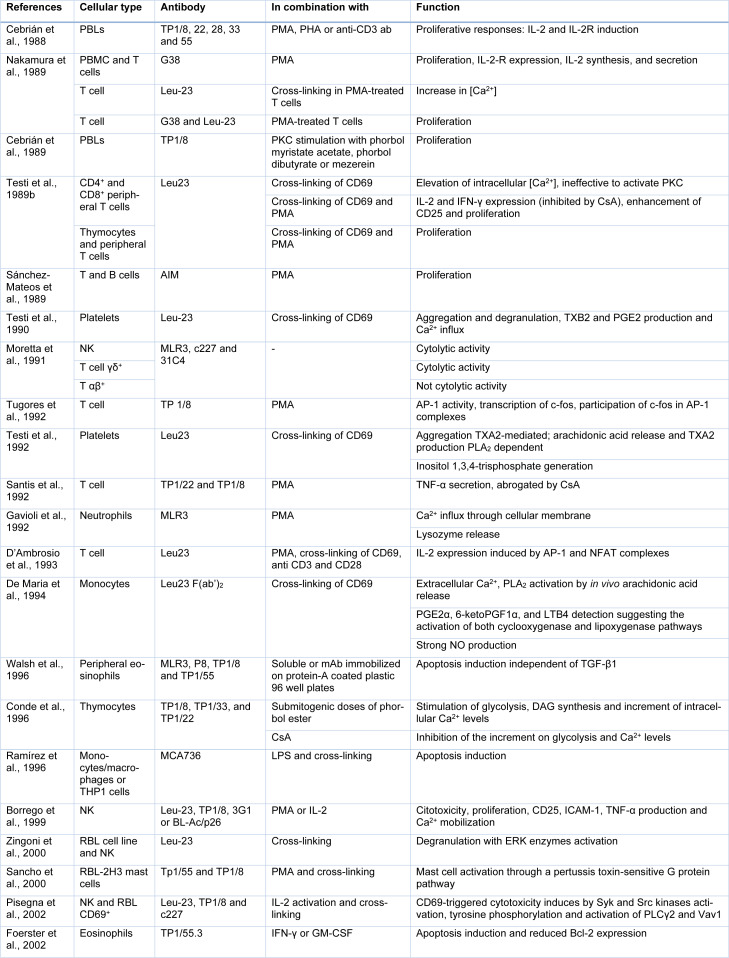
*In vitro* studies employing anti-CD69 monoclonal antibodies in different human cells. Abbreviations included in Table 1. Peripheral blood lymphocytes, PBLs; peripheral blood mononuclear cells, PBMCs; Natural killer cells, NK; Phorbol 12-myristate 13-acetate, PMA; Phytohemagglutinin, PHA; Protein kinase C, PKC; Interferon-γ, IFN-γ; Nuclear factor of activated T cells 1, NFAT; Thromboxane B2, TXB2; Thromboxane A2, TXA2; Prostaglandin E2, PGE2; Activator protein 1, AP-1; Phospholipase A2, PLA_2_; Cyclosporine A, CsA; Lipopolysaccharide, LPS; Granulocyte macrophage colony-stimulating factor ,GM-CSF; 6-keto-prostaglandin F1α, 6-ketoPGF1α; Leukotriene B4, LTB4; Nitric oxide, NO; Transforming growth factor-beta 1,TGF-β1; Diacylglycerol, DAG; intercellular adhesion molecule-1, ICAM-1; tumor necrosis factor, TNF-α; extracellular signal-regulated kinase, ERK; Tyrosine-protein kinase, Syk; Proto-oncogene tyrosine-protein kinase, Src; Phospholipase Cγ2, PLCγ2; Vav Guanine Nucleotide Exchange Factor 1, Vav1

## References

[1] Bankovich AJ, Shiow LR, Cyster JG (2010). CD69 suppresses sphingosine 1-phosophate receptor-1 (S1P1) function through interaction with membrane helix 4. J Biol Chem.

[2] Blanco-Domínguez R, de la Fuente H, Rodríguez C, Martín-Aguado L, Sánchez-Díaz R, Jiménez-Alejandre R (2022). CD69 expression on regulatory T cells protects from immune damage after myocardial infarction. J Clin Invest. 2022;132. Clin Invest.

[3] Borrego F, Robertson MJ, Ritz J, Peña J, Solana R (1999). CD69 is a stimulatory receptor for natural killer cell and its cytotoxic effect is blocked by CD94 inhibitory receptor. Immunology.

[4] Bu DX, Tarrio M, Maganto-Garcia E, Stavrakis G, Tajima G, Lederer J (2011). Impairment of the programmed cell death-1 pathway increases atherosclerotic lesion development and inflammation. Arterioscler Thromb Vasc Biol.

[5] Cebrián M, Redondo JM, López-Rivas A, Rodríguez-Tarduchy G, De Landázuri MO, Sánchez-Madrid F (1989). Expression and function of AIM, an activation inducer molecule of human lymphocytes, is dependent on the activation of protein kinase C. Eur J Immunol.

[6] Cebrián M, Yagüe E, Rincón M, López-Botet M, de Landázuri MO, Sánchez-Madrid F (1988). Triggering of T cell proliferation through AIM, an activation inducer molecule expressed on activated human lymphocytes. J Exp Med.

[7] Chiba K (2005). FTY720, a new class of immunomodulator, inhibits lymphocyte egress from secondary lymphoid 7tissues and thymus by agonistic activity at sphingosine 1-phosphate receptors. Pharmacol Ther.

[8] Cibrián D, Saiz ML, De La Fuente H, Sánchez-Díaz R, Moreno-Gonzalo O, Jorge I (2016). CD69 controls the uptake of L-tryptophan through LAT1-CD98 and AhR-dependent secretion of IL-22 in psoriasis. Nat Immunol.

[9] Cibrián D, Sánchez-Madrid F (2017). Europe PMC Funders Group CD69 : from activation marker to metabolic gatekeeper. Eur J Immunol.

[10] Conde M, Montaño R, Moreno-Aurioles VR, Ramirez R, Sanchez-Mateos P, Sanchez-Madrid F (1996). Anti-CD69 antibodies enhance phorbol-dependent glucose metabolism and Ca2+ levels in human thymocytes. Antagonist effect of cyclosporin A. J Leukoc Biol.

[11] Cortés JR, Sánchez-Díaz R, Bovolenta ER, Barreiro O, Lasarte S, Matesanz-Marín A (2014). Maintenance of immune tolerance by Foxp3+ regulatory T cells requires CD69 expression. J Autoimmun.

[12] Cosulich ME, Rubartelli A, Risso A, Cozzolino F, Bargellesi A (1987). Functional characterization of an antigen involved in an early step of T-cell activation. Proc Natl Acad Sci U S A.

[13] Cruz-Adalia A, Jiménez-Borreguero LJ, Ramírez-Huesca M, Chico-Calero I, Barreiro O, López-Conesa E (2010). CD69 limits the severity of cardiomyopathy after autoimmune myocarditis. Circulation.

[14] D’Ambrosio D, Trotta R, Vacca A, Frati L, Santoni A, Gulino A (1993). Transcriptional regulation of interleukin‐2 gene expression by CD69‐generated signals. Eur J Immunol.

[15] Dang EV, Barbi J, Yang HY, Jinasena D, Yu H, Zheng Y (2011). Control of T(H)17/T(reg) balance by hypoxia-inducible factor 1. Cell.

[16] de la Fuente H, Cruz-Adalia A, Martinez del Hoyo G, Cibrián-Vera D, Bonay P, Pérez-Hernández D (2014). The leukocyte activation receptor CD69 controls T cell differentiation through its interaction with galectin-1. Mol Cell Biol.

[17] de Maria R, Cifone MG, Trotta R, Rippo MR, Festuccia C, Santoni A (1994). Triggering of human monocyte activation through CD69, a member of the natural killer cell gene complex family of signal transducing receptors. J Exp Med.

[18] Esplugues E, Sancho D, Vega-Ramos J, Martínez-A C, Syrbe U, Hamann A (2003). Enhanced antitumor immunity in mice deficient in CD69. J Exp Med.

[19] Fernandez DM, Rahman AH, Fernandez NF, Chudnovskiy A, Amir ED, Amadori L (2019). Single-cell immune landscape of human atherosclerotic plaques. Nat Med.

[20] Fernández-Gallego N, Sánchez-Madrid F, Cibrian D (2021). Role of AHR ligands in skin homeostasis and cutaneous inflammation. Cells.

[21] Foerster M, Haefner D, Kroegel C (2002). Bcl-2-mediated regulation of CD69-induced apoptosis of human eosinophils: identification and characterization of a novel receptor-induced mechanism and relationship to CD95-transduced signalling. Scand J Immunol.

[22] Fucikova J, Spisek R, Kroemer G, Galluzzi L (2020). Calreticulin and cancer. Cell Res.

[23] Gavioli R, Risso A, Smilovich D, Baldissarro I, Capra MC, Bargellesi A (1992). CD69 molecule in human neutrophils: Its expression and role in signal-transducing mechanisms. Cell Immunol.

[24] Glal D, Sudhakar JN, Lu HH, Liu MC, Chiang HY, Liu YC (2018). ATF3 sustains IL-22-induced STAT3 phosphorylation to maintain mucosal immunity through inhibiting phosphatases. Front Immunol.

[25] González-Amaro R, Cortés JR, Sánchez-Madrid F, Martín P (2013). Is CD69 an effective brake to control inflammatory diseases?. Trends Mol Med.

[26] Grievink HW, Smit V, Verwilligen RAF, Kleijn MNAB, Smeets D, Binder CJ (2021). Stimulation of the PD-1 pathway decreases atherosclerotic lesion development in ldlr deficient mice. Front Cardiovasc Med.

[27] Hansson GK, Libby P (2006). The immune response in atherosclerosis: a double-edged sword. Nat Rev Immunol.

[28] Hara T, Jung LK, Bjorndahl JM, Fu SM (1986). Human T cell activation. III. Rapid induction of a phosphorylated 28 kD/32 kD disulfide-linked early activation antigen (EA 1) by 12-o-tetradecanoyl phorbol-13-acetate, mitogens, and antigens. J Exp Med.

[29] Hare KJ, Jenkinson EJ, Anderson G (1999). CD69 expression discriminates MHC-dependent and -independent stages of thymocyte positive selection. J Immunol.

[30] Hasegawa A, Iwamura C, Kitajima M, Hashimoto K, Otsuyama K, Ogino H (2013). Crucial role for CD69 in the pathogenesis of dextran sulphate sodium-induced colitis. PLoS One.

[31] Hayashizaki K, Kimura MY, Tokoyoda K, Hosokawa H, Shinoda K, Hirahara K (2016). Myosin light chains 9 and 12 are functional ligands for CD69 that regulate airway inflammation. Sci Immunol.

[32] Jiménez-Fernández M, Rodríguez-Sinovas C, Cañes L, Ballester-Servera C, Vara A, Requena S (2022). CD69-oxLDL ligand engagement induces Programmed Cell Death 1 (PD-1) expression in human CD4 + T lymphocytes. Cell Mol Life Sci.

[33] Kimura MY, Igi A, Hayashizaki K, Mita Y, Shinzawa M, Kadakia T (2018). CD69 prevents PLZFhi innate precursors from prematurely exiting the thymus and aborting NKT2 cell differentiation. Nat Commun.

[34] Kopf H, de la Rosa GM, Howard OMZ, Chen X (2007). Rapamycin inhibits differentiation of Th17 cells and promotes generation of FoxP3+ T regulatory cells. Int Immunopharmacol.

[35] Labiano S, Meléndez-Rodríguez F, Palazón A, Teijeira Á, Garasa S, Etxeberria I (2017). CD69 is a direct HIF-1α target gene in hypoxia as a mechanism enhancing expression on tumor-infiltrating T lymphocytes. Oncoimmunology. 2017;6. Oncoimmunology.

[36] Lamana A, Martin P, De La Fuente H, Martinez-Mũoz L, Cruz-Adalia A, Ramirez-Huesca M (2011). CD69 modulates sphingosine-1-phosphate-induced migration of skin dendritic cells. J Invest Dermatol.

[37] Lanier LL, Buck DW, Rhodes L, Ding A, Evans E, Barney C (1988). Interleukin 2 activation of natural killer cells rapidly induces the expression and phosphorylation of the Leu-23 activation antigen. J Exp Med.

[38] Lauzurica P, Sancho D, Torres M, Albella B, Marazuela M, Merino T (2000). Phenotypic and functional characteristics of hematopoietic cell lineages in CD69-deficient mice. Blood.

[39] Lin CR, Wei TYW, Tsai HY, Wu YT, Wu PY, Chen ST (2015). Glycosylation-dependent interaction between CD69 and S100A8/S100A9 complex is required for regulatory T-cell differentiation. FASEB J.

[40] López-Cabrera M, Santis AG, Fernández-Ruiz E, Blacher R, Esch F, Sánchez-Mateos P (1993). Molecular cloning, expression, and chromosomal localization of the human earliest lymphocyte activation antigen AIM/CD69, a new member of the C-Type animal lectin superfamily of signal-transmitting receptors. J Exp Med.

[41] Mackay LK, Braun A, Macleod BL, Collins N, Tebartz C, Bedoui S (2015). Cutting edge: CD69 interference with sphingosine-1-phosphate receptor function regulates peripheral T cell retention. J Immunol.

[42] Manié S, Kubar J, Limouse M, Ferrua B, Ticchioni M, Breittmayer JP (1993). CD3-stimulated Jurkat T cells mediate IL-1 beta production in monocytic THP-1 cells. Role of LFA-1 molecule and participation of CD69 T cell antigen. Eur Cytokine Netw.

[43] Martín P, Gómez M, Lamana A, Cruz-Adalia A, Ramírez-Huesca M, Ursa MÁ (2010). CD69 association with Jak3/Stat5 proteins regulates Th17 cell differentiation. Mol Cell Biol.

[44] Martín P, Gómez M, Lamana A, Marín AM, Cortés JR, Ramírez-Huesca M (2010). The leukocyte activation antigen CD69 limits allergic asthma and skin contact hypersensitivity. J Allergy Clin Immunol.

[45] Martín-Gayo E, Sierra-Filardi E, Corbí AL, Toribio ML (2010). Plasmacytoid dendritic cells resident in human thymus drive natural Treg cell development. Blood.

[46] Mascanfroni ID, Takenaka MC, Yeste A, Patel B, Wu Y, Kenison JE (2015). Metabolic control of type 1 regulatory T cell differentiation by AHR and HIF1-α. Nat Med.

[47] Matloubian M, Lo CG, Cinamon G, Lesneski MJ, Xu Y, Brinkmann V (2004). Lymphocyte egress from thymus and peripheral lymphoid organs is dependent on S1P receptor 1. Nature.

[48] Mcinnes IB, Leung BP, Sturrock RD, Field M, Liew FY (1997). Interleukin-15 mediates T cell-dependent regulation of tumor necrosis factor-alpha production in rheumatoid arthritis. Nat Med.

[49] Mita Y, Kimura MY, Hayashizaki K, Koyama-Nasu R, Ito T, Motohashi S (2018). Crucial role of CD69 in anti-tumor immunity through regulating the exhaustion of tumor-infiltrating T cells. Int Immunol.

[50] Moretta A, Poggi A, Pende D, Tripodi G, Orengo AM, Pella N (1991). CD69-mediated pathway of lymphocyte activation: anti-CD69 monoclonal antibodies trigger the cytolytic activity of different lymphoid effector cells with the exception of cytolytic T lymphocytes expressing T cell receptor alpha/beta. J Exp Med.

[51] Nakamura S, Sung SS, Bjorndahl JM, Fu SM (1989). Human T cell activation. IV. T cell activation and proliferation via the early activation antigen EA 1. J Exp Med.

[52] Notario L, Alari-Pahissa E, Albentosa A, Leiva M, Sabio G, Lauzurica P (2018). Anti-CD69 therapy induces rapid mobilization and high proliferation of HSPCs through S1P and mTOR. Leukemia.

[53] Odagiu L, May J, Boulet S, Baldwin TA, Labrecque N (2021). Role of the orphan nuclear receptor NR4A family in T-cell biology. Front Endocrinol (Lausanne).

[54] Oh H, Ghosh S (2013). NF-κB: roles and regulation in different CD4(+) T-cell subsets. Immunol Rev.

[55] Pisegna S, Zingoni A, Pirozzi G, Cinque B, Cifone MG, Morrone S (2002). Src-dependent Syk activation controls CD69-mediated signaling and function on human NK cells. J Immunol.

[56] Quintana FJ, Basso AS, Iglesias AH, Korn T, Farez MF, Bettelli E (2008). Control of T(reg) and T(H)17 cell differentiation by the aryl hydrocarbon receptor. Nature.

[57] Radulovic K, Manta C, Rossini V, Holzmann K, Kestler HA, Wegenka UM (2012). CD69 regulates type I IFN-induced tolerogenic signals to mucosal CD4 T cells that attenuate their colitogenic potential. J Immunol.

[58] Radulovic K, Rossini V, Manta C, Holzmann K, Kestler HA, Niess H (2013). The early activation marker CD69 regulates the expression of chemokines and CD4 T cell accumulation in intestine. PLoS One.

[59] Ramírez R, Carracedo J, Castedo M, Zamzami N, Kroemer G (1996). CD69-induced monocyte apoptosis involves multiple nonredundant signaling pathways. Cell Immunol.

[60] Risso A, Smilovich D, Capra MC, Baldissarro I, Yan G, Bargellesi A (1991). CD69 in resting and activated T lymphocytes. Its association with a GTP binding protein and biochemical requirements for its expression. J Immunol.

[61] Russo A, Schürmann H, Brandt M, Scholz K, Matos ALL, Grill D (2022). Alarming and calming: opposing roles of S100A8/S100A9 dimers and tetramers on monocytes. Adv Sci (Weinh).

[62] Sánchez-Díaz R, Blanco-Dominguez R, Lasarte S, Tsilingiri K, Martín-Gayo E, Linillos-Pradillo B (2017). Thymus-derived regulatory T cell development is regulated by C-Type lectin-mediated BIC/microRNA 155 expression. Mol Cell Biol.

[63] Sánchez-Mateos P, Cebrián M, Acevedo A, López-Botet M, De Landázuri MO, Sánchez-Madrid F (1989). Expression of a gp33/27,000 MW activation inducer molecule (AIM) on human lymphoid tissues. Induction of cell proliferation on thymocytes and B lymphocytes by anti-AIM antibodies. Immunology.

[64] Sancho D, Gómez M, Viedma F, Esplugues E, Gordón-Alonso M, Angeles García-López M (2003). CD69 downregulates autoimmune reactivity through active transforming growth factor-beta production in collagen-induced arthritis. J Clin Invest.

[65] Sancho D, Santis AG, Alonso-Lebrero JL, Viedma F, Tejedor R, Sánchez-Madrid F (2000). Functional analysis of ligand-binding and signal transduction domains of CD69 and CD23 C-type lectin leukocyte receptors. J Immunol.

[66] Santis AG, Campanero MR, Alonso JL, Tugores A, Alonso MA, Yagüe E (1992). Tumor necrosis factor-α production induced in T lymphocytes through the AIM/CD69 activation pathway. Eur J Immunol.

[67] Scalise M, Galluccio M, Console L, Pochini L, Indiveri C (2018). The human SLC7A5 (LAT1): the intriguing histidine/large neutral amino acid transporter and its relevance to human health. Front Chem.

[68] Sekiya T, Kashiwagi I, Yoshida R, Fukaya T, Morita R, Kimura A (2013). Nr4a receptors are essential for thymic regulatory T cell development and immune homeostasis. Nat Immunol.

[69] Shiow LR, Rosen DB, Brdičková N, Xu Y, An J, Lanier LL (2006). CD69 acts downstream of interferon-alpha/beta to inhibit S1P1 and lymphocyte egress from lymphoid organs. Nature.

[70] Sinclair LV (2013). , Rolf J, Emslie E, Shi YB, Taylor PM, Cantrell DA. Antigen receptor control of amino acid transport coordinates the metabolic re-programming that is essential for T cell differentiation. Nat Immunol.

[71] Skon CN, Lee JY, Anderson KG, Masopust D, Hogquist KA, Jameson SC (2013). Transcriptional downregulation of S1pr1 is required for the establishment of resident memory CD8+ T cells. Nat Immunol.

[72] Srikrishna G, Panneerselvam K, Westphal V, Abraham V, Varki A, Freeze HH (2001). Two proteins modulating transendothelial migration of leukocytes recognize novel carboxylated glycans on endothelial cells. J Immunol.

[73] Steinbach K, Vincenti I, Merkler D (2018). Resident-memory T Cells in tissue-restricted immune responses: For better or worse? Front Immunol.

[74] Sundblad V, Morosi LG, Geffner JR, Rabinovich GA (2017). Galectin-1: A Jack-of-All-Trades in the Resolution of acute and chronic inflammation. J Immunol.

[75] Tan C, Hiwa R, Mueller JL, Vykunta V, Hibiya K, Noviski M (2020). NR4A nuclear receptors restrain B cell responses to antigen when second signals are absent or limiting. Nat Immunol.

[76] Testi R, Phillips JH, Lanier LL (1988). Constitutive expression of A phosphorylated activation antigen (Leu 23) by CD3(bright) human thymocytes. J Immunol.

[77] Testi R, Phillips JH, Lanier LL (1989a). Leu 23 induction as an early marker of functional CD3/T cell antigen receptor triggering. Requirement for receptor cross-linking, prolonged elevation of intracellular. J Immunol.

[78] Testi R, Phillips JH, Lanier LL (1989b). T cell activation via Leu-23 (CD69). J Immunol.

[79] Testi R, Pulcinelli F, Frati L, Gazzaniga PP, Santoni A (1990). CD69 is expressed on platelets and mediates platelet activation and aggregation. J Exp Med.

[80] Testi R, Pulcinelli FM, Cifone MG, Botti D, Del Grosso E, Riondino S (1992). Preferential involvement of a phospholipase A2-dependent pathway in CD69-mediated platelet activation. J Immunol.

[81] Tsilingiri K, De La Fuente H, Relaño M, Sánchez-Díaz R, Rodríguez C, Crespo J (2019). Oxidized Low-density lipoprotein receptor in lymphocytes prevents atherosclerosis and predicts subclinical disease. Circulation.

[82] Tugores A, Alonso MA, Sánchez-Madrid F, de Landázuri MO (1992). Human T cell activation through the activation-inducer molecule/CD69 enhances the activity of transcription factor AP-1. J Immunol.

[83] Vance BA, Harley PH, Backlund PS, Ward Y, Phelps TL, Gress RE (2005). Human CD69 associates with an N-terminal fragment of calreticulin at the cell surface. Arch Biochem Biophys.

[84] Vance BA, Wu W, Ribaudo RK, Segal DM, Kearse KP (1997). Multiple dimeric forms of human CD69 result from differential addition of n-glycans to typical (Asn-X-Ser/Thr) and Atypical (Asn-X-Cys) glycosylation motifs. J Biol Chem.

[85] Walsh GM, Williamson ML, Symon FA, Willars GB, Wardlaw AJ (1996). Ligation of CD69 induces apoptosis and cell death in human eosinophils cultured with granulocyte-macrophage colony-stimulating factor. Blood.

[86] Zenewicz LA (2018). IL-22: There is a gap in our knowledge. ImmunoHorizons.

[87] Ziegler SF, Ramsdell F, Hjerrild KA, Armitage RJ, Grabstein KH, Hennen KB (1993). Molecular characterization of the early activation antigen CD69: A type II membrane glycoprotein related to a family of natural killer cell activation antigens. Eur J Immunol.

[88] Zingoni A, Palmieri G, Morrone S, Carretero M, Lopez-Botel M, Piccoli M (2000). CD69-triggered ERK activation and functions are negatively regulated by CD94 / NKG2-A inhibitory receptor. Eur J Immunol.

